# Supplementary material for: Naked mole-rat brown fat thermogenesis is diminished during hypoxia through a rapid decrease in UCP1
Source: Nat Commun. 2021 Nov 23;12:6801. doi: 10.1038/s41467-021-27170-2 (PMC8610999; doi:10.1038/s41467-021-27170-2)
Supplement: Supplementary file 1 — Supplementary information. [file 41467_2021_27170_MOESM1_ESM.pdf]

Supplementary Information

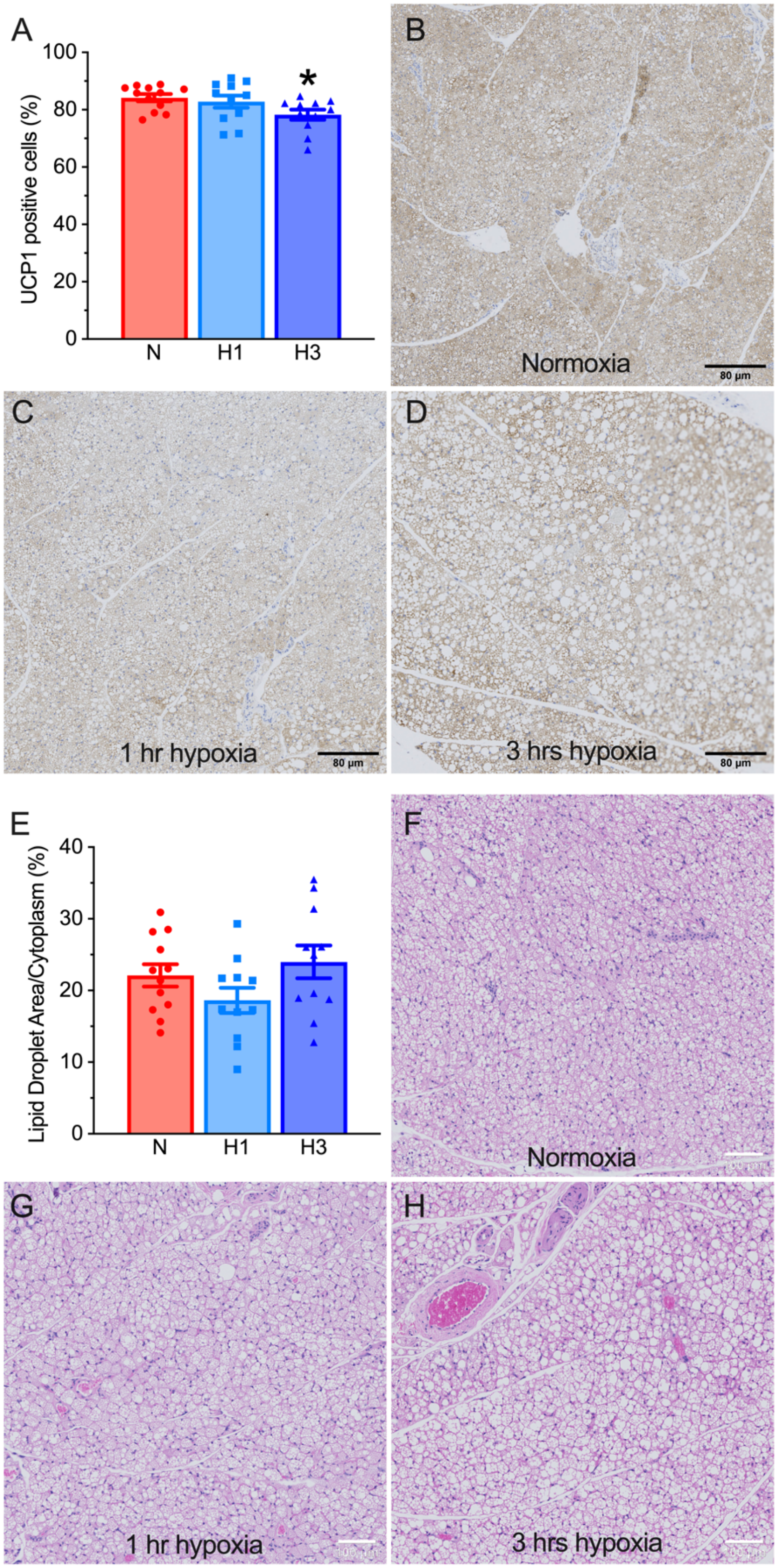

Figure S1. Immunohistochemistry analysis of uncoupling protein 1 (UCP1) and lipid droplets.

UCP1-positive cell number and lipid droplet (LD) area do not change markedly with acute hypoxia in naked mole-rat brown adipose tissue (BAT). (A) Summary of UCP1-positive cells in interscapular BAT from naked mole-rats treated in normoxia (21% O_2; red bars; n = 12), or 1 or 3 hrs of hypoxia (7% O_2; light and dark blue bars, respectively; n = 11 each). (B-D) Representative images of UCP1 staining in BAT cells from naked mole-rats treated as in A. (E) Summary of LD area relative to total cytoplasmic area in interscapular BAT from naked mole-rats treated in normoxia (21% O_2; n = 12), or 1 or 3 hrs of hypoxia (7% O_2; n = 11 each). (F-H) Representative images of hematoxylin and eosin (H&E) staining in BAT from naked mole-rats treated as in E. Data are mean ± SEM. Asterisks indicate significant difference from normoxic controls (One-sided Welch's ANOVA test with Dunnett's post-test; $F_{2,34} = 1.915$, $p = 0.1734$ for LD, $F_{2,34} = 3.684$, $p = 0.0442$ for UCP1). Abbreviations: N – normoxia, H1 – 1hr hypoxia, H3 – 3 hrs hypoxia. Source data are provided as a Source Data file.

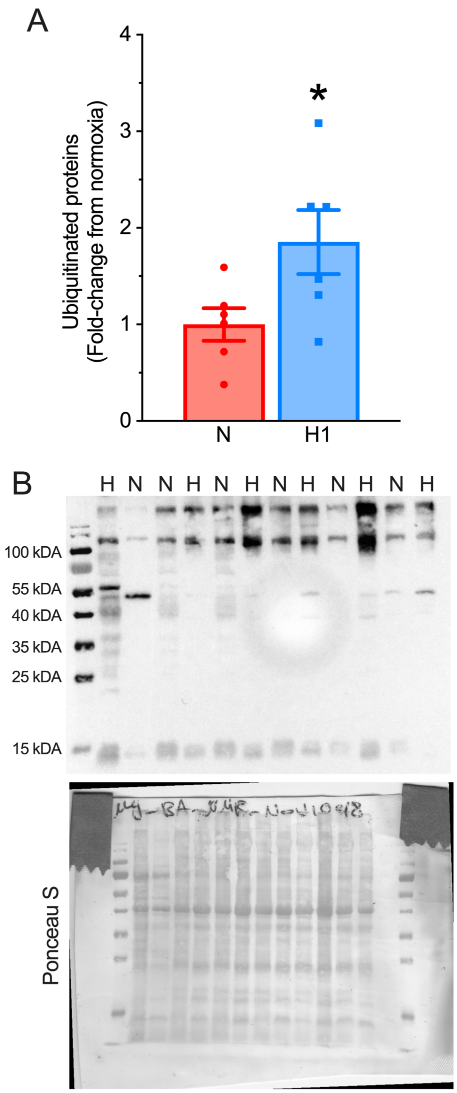

Figure. S2. Acute hypoxia increases protein ubiquitination in naked mole-rat BAT.

(A) Summary of western blot analysis of protein ubiquitination levels in interscapular BAT homogenates from naked mole-rats held at 30°C in normoxia (21% O_2, red bar; n = 6) or after 1 hr of hypoxia (7% O_2, light blue bar; n = 6). (B) Western blot image of ubiquitinated protein bands and total protein gel expression quantified with Ponceau S staining. Data are mean ± SEM. Asterisks indicate significant difference from normoxic controls (paired two-tailed Student's t-test; p = 0.0312). Abbreviations: N – normoxia, H1 – 1hr hypoxia, H –hypoxia. Source data are provided as a Source Data file.
