# Supplementary material for: Naked mole-rat brown fat thermogenesis is diminished during hypoxia through a rapid decrease in UCP1
Source: Nat Commun. 2021 Nov 23;12:6801. doi: 10.1038/s41467-021-27170-2 (PMC8610999; doi:10.1038/s41467-021-27170-2)
Supplement: Supplementary file 3 — Description of Additional Supplementary Files. [file 41467_2021_27170_MOESM3_ESM.docx]

Description of Additional Supplementary Files

Title: Supplementary Movie 1.

Description: Time-lapse thermal video of a naked mole-rat during a normoxic 🡪 hypoxic 🡪 normoxic transition.

Title: Supplementary Movie 2.

Description: Time-lapse thermal video of a naked mole-rat during a normoxic 🡪 hypoxic 🡪 normoxic transition following isoproterenol injection.
