# Supplementary material for: Naked mole-rat brown fat thermogenesis is diminished during hypoxia through a rapid decrease in UCP1
Source: Nat Commun. 2021 Nov 23;12:6801. doi: 10.1038/s41467-021-27170-2 (PMC8610999; doi:10.1038/s41467-021-27170-2)
Supplement: Supplementary file 6 — Reporting summary. [file 41467_2021_27170_MOESM6_ESM.pdf]

nature portfolio

Corresponding author(s): Matthew Pamenter

Last updated by author(s): YYYY-MM-DD

Reporting Summary

Nature Portfolio wishes to improve the reproducibility of the work that we publish. This form provides structure for consistency and transparency in reporting. For further information on Nature Portfolio policies, see our Editorial Policies and the Editorial Policy Checklist.

Statistics

For all statistical analyses, confirm that the following items are present in the figure legend, table legend, main text, or Methods section.

n/a	Confirmed	
☐	☒	The exact sample size (n) for each experimental group/condition, given as a discrete number and unit of measurement
☐	☒	A statement on whether measurements were taken from distinct samples or whether the same sample was measured repeatedly
☐	☒	The statistical test(s) used AND whether they are one- or two-sided<br>*Only common tests should be described solely by name; describe more complex techniques in the Methods section.*
☐	☒	A description of all covariates tested
☐	☒	A description of any assumptions or corrections, such as tests of normality and adjustment for multiple comparisons
☐	☒	A full description of the statistical parameters including central tendency (e.g. means) or other basic estimates (e.g. regression coefficient) AND variation (e.g. standard deviation) or associated estimates of uncertainty (e.g. confidence intervals)
☐	☒	For null hypothesis testing, the test statistic (e.g. F, t, r) with confidence intervals, effect sizes, degrees of freedom and P value noted<br>*Give P values as exact values whenever suitable.*
☒	☐	For Bayesian analysis, information on the choice of priors and Markov chain Monte Carlo settings
☒	☐	For hierarchical and complex designs, identification of the appropriate level for tests and full reporting of outcomes
☒	☐	Estimates of effect sizes (e.g. Cohen's d, Pearson's r), indicating how they were calculated

Our web collection on statistics for biologists contains articles on many of the points above.

Software and code

Policy information about availability of computer code

Data collection	Thermal imaging data was collected and analyzed using Thermacam Research Pro V2.9 (Teledyne FLIR, LLC, Wilsonville, OR, USA) and the Thermimage package V3.0 in R to derive correction factors. Immunohistochemistry images were imaged using Mirax Viewer Image software (V1.6, Carl Zeiss Canada Ltd. Toronto, Canada) and extracted using Aperio ImageScope software (V12.3.3; Leica Biosystems, Buffalo Grove, Ill, USA).
Data analysis	Forward-looking infrared (FLIR) thermal images were analyzed using a computerized acquisition program (Thermacam Researcher Pro V2.9, Teledyne). Western blot and transmission electron microscopy files were analyzed using ImageJ software (Version 1.53j, NIH, Bethesda, MD). Immunohistochemistry images were processed using Zeiss software ZEN 3.2 (Zen Lite; Carl Zeiss Canada Ltd.). Statistical analysis were performed using Graphpad Prism 9 (Graphpad Prism, La Jolla, CA, USA).

For manuscripts utilizing custom algorithms or software that are central to the research but not yet described in published literature, software must be made available to editors and reviewers. We strongly encourage code deposition in a community repository (e.g. GitHub). See the Nature Portfolio guidelines for submitting code & software for further information.

Data

Policy information about availability of data

All manuscripts must include a data availability statement. This statement should provide the following information, where applicable:

- Accession codes, unique identifiers, or web links for publicly available datasets
- A description of any restrictions on data availability
- For clinical datasets or third party data, please ensure that the statement adheres to our policy

All data generated in this study have been deposited in the Figshare database: https://figshare.com/s/e5e7767403970f6285de, https://figshare.com/

March 2021

s/7e40f33306fae3289e0c, https://figshare.com/s/2a77c3c04db76b5a25d1, and https://figshare.com/s/9e1bdce1f4538252c474.

Field-specific reporting

Please select the one below that is the best fit for your research. If you are not sure, read the appropriate sections before making your selection.

- [x] Life sciences
- [ ] Behavioural & social sciences
- [ ] Ecological, evolutionary & environmental sciences

For a reference copy of the document with all sections, see nature.com/documents/nr-reporting-summary-flat.pdf

Life sciences study design

All studies must disclose on these points even when the disclosure is negative.

Sample size	Sample sizes were chosen based on our extensive previous experience conducting previous experiments using similar techniques and in these species and are consistent with or exceed the standards in our field for measurements from small animals<br>https://royalsocietypublishing.org/doi/10.1098/rspb.2019.0841<br>https://journals.plos.org/plosone/article?id=10.1371/journal.pone.0040933<br>https://www.sciencedirect.com/science/article/pii/S221287781830958X?via%3Dihub
Data exclusions	1- Membrane#1 for UCP1 and OXPHOS-NMR blots: We excluded the last two lanes (normoxia and hypoxia BAT samples) because they were either contaminated with WAT or they contained more fatty tissues.<br><br>2- CHP membrane: We excluded the first lane in the normoxia condition for OXPHOS blot because we mistakingly loaded the wrong sample (mouse sample).
Replication	All physiological experiments were repeated a minimum of 6 times using different, randomly chosen animals for all treatments reported herein. Western blot and IHC analyses was repeated in separate batches of samples and gels were run multiple times to ensure reproducibility from the same sample. Western blots were run on 2-4 separate blots with independent biological replicates on each blot. IHC analysis was repeated in 2 separate sets of independent biological replicates. Electron microscopy data was repeated from several animals with multiple slices evaluated from each animal. All electron microscopy experiments were conducted simultaneously. All replications were successful for all experimental approaches.
Randomization	Lab-raised animals were randomly selected from different colonies for all experiments. Wild-caught animals were randomly selected due to the nature of trapping live animals and were randomly divided into normoxic and hypoxic treatment groups. Physiological experiments were conducted in random order and repeated over several weeks in different animals. Sham and drug injection treatments were also randomized.
Blinding	For the thermal imaging experiments, different researchers performed the experiments and analyzed the data independently of each other, with the analysis conducted blindly to the expected impact of each treatment. Thermal imaging experiments were not conducted in a blinded fashion because they were performed in thermally stable rooms set at the various experimental temperatures, and thus the treatments were clearly evident to the researcher within each environment. Electron microscopy experiments and IHC analysis were similarly performed in a blinded fashion with the analyzing collaborator unaware of the treatment for each sample. Western blot experiments were not blinded because it was necessary to load wells in a known order for analysis and because Western blot analysis is minimally susceptible to bias because of the use of loading controls and consistent measurements of signal intensity from samples loaded in the same gel.

Reporting for specific materials, systems and methods

We require information from authors about some types of materials, experimental systems and methods used in many studies. Here, indicate whether each material, system or method listed is relevant to your study. If you are not sure if a list item applies to your research, read the appropriate section before selecting a response.

Materials & experimental systems

n/a	Involved in the study	
[ ]	[x]	Antibodies
[x]	[ ]	Eukaryotic cell lines
[x]	[ ]	Palaeontology and archaeology
[ ]	[x]	Animals and other organisms
[x]	[ ]	Human research participants
[x]	[ ]	Clinical data
[x]	[ ]	Dual use research of concern

Methods

n/a	Involved in the study	
[x]	[ ]	ChIP-seq
[x]	[ ]	Flow cytometry
[x]	[ ]	MRI-based neuroimaging

Antibodies

Antibodies used	UCP1 antibody (Sigma Aldrich, #U6382), total oxidative phosphorylation (OXPHOS) rodent cocktail (Abcam, #Ab110413), anti-ubiquitin (Abcam, #Ab7780), LC3B (Cell Signaling Technology, #2775), PARKIN (ABclonal, #A0968), p62 (ABclonal, #A11483), PINK1

(ABclonal, #A7131), FUNDC1 (ABclonal, #A16318), BNIP3 (ABcam, #Ab10433), BNIP3L (Santa Cruz, #SC-166314), UCP3 (ABcam, #AB3477), Fis1 (BioVision, #3491), Drp1 (BD Biosciences, #611113), Drp1-S616 (ABclonal, #AP0849), Drp1-S637 (Cell Signaling Technology, #4867), Casp3 (ABclonal, #A0214), p53 (Cell Signalling Technology, #2524), AIF (Abcam, #ab32516), BAX (Santa Cruz, #SC-526), Bcl2 (Santa Cruz, #sc-492), Mff (ABclonal, #A4874), Opa1 (ABclonal, #A9833), Mfn1 (ABclonal, #A9880), Mfn2 (ABclonal," "#A19678), TOM20 (ABclonal, #A19403), TBC1D15 (ABclonal, #A10593), and RAB7A (ABclonal, #A12784).

Validation

UCP1, https://www.sigmaaldrich.com/catalog/product/sigma/u6382?lang=en&region=CA
OXPHOS, https://www.abcam.com/total-oxphos-rodent-wb-antibody-cocktail-ab110413.html
Anti-Ubiquitin, https://www.abcam.com/ubiquitin-antibody-ab7780.html
LC3B, https://www.cellsignal.com/products/primary-antibodies/lc3b-antibody/2775
PARKIN, https://abclonal.com/catalog-antibodies/ParkinRabbitpAb/A0968
p62, https://abclonal.com/catalog-antibodies/KOValidatedSQSTM1p62RabbitpAb/A11483
PINK1, https://abclonal.com/catalog-antibodies/PINK1RabbitpAb/A7131
FUNDC1, https://abclonal.com/catalog-antibodies/FUNDC1RabbitpAb/A16318
BNIP3, https://www.abcam.com/bnip3-antibody-ana40-ab10433.html
BNIP3L, https://www.scbt.com/p/nix-antibody-e-1?productCanUrl=nix-antibody-e-1&_requestid=1879361
UCP3, https://www.abcam.com/ucp3-antibody-ab3477.html
Fis1, https://www.biovision.com/fis1-antibody.html
Drp1, https://www.bdbiosciences.com/en-us/products/reagents/microscopy-imaging-reagents/immunofluorescence-reagents/purified-mouse-anti-dlp1.611113
Drp1-S616, https://abclonal.com/catalog-antibodies/PhosphoDRP1S616RabbitpAb/AP0849
Drp1-S637, https://www.cellsignal.com/products/primary-antibodies/phospho-drp1-ser637-antibody/4867
Casp3, https://abclonal.com/catalog-antibodies/Caspase3RabbitpAb/A0214
p53, https://www.cellsignal.com/products/primary-antibodies/p53-1c12-mouse-mab/2524
AIF, https://www.abcam.com/aif-antibody-e20-mitochondrial-marker-ab32516.html
BAX, https://www.scbt.com/p/bax-antibody-p-19?requestFrom=search
Bcl2, https://www.scbt.com/p/bcl-2-antibody-n-19?requestFrom=search
Mff, https://abclonal.com/catalog-antibodies/MFFPolyclonalAntibody/A4874
Opa1, https://abclonal.com/catalog-antibodies/OPA1RabbitpAb/A9833
Mfn1, https://abclonal.com/catalog-antibodies/MFN1PolyclonalAntibody/A9880
Mfn2, https://abclonal.com/catalog-antibodies/Mitofusin2RabbitmAb/A19678
TOM20, https://abclonal.com/catalog-antibodies/TOM20RabbitmAb/A19403
TBC1D15, https://abclonal.com/catalog-antibodies/TBC1D15PolyclonalAntibody/A10593
RAB7A, https://abclonal.com/catalog-antibodies/RAB7APolyclonalAntibody/A12784

Animals and other organisms

Policy information about studies involving animals; ARRIVE guidelines recommended for reporting animal research

Laboratory animals

Adult (1-2 year old) subordinate male and female naked mole-rats (Heterocephalus glaber, 135 Adult, 47.3 ± 3.1g, all subordinate, random sex.) were bred and housed at the University of Ottawa. Naked mole-rats were bred at the University of Ottawa and group-housed in interconnected multi-cage systems at 30°C and 21% O2 in 50% humidity with a 12L:12D light cycle. Animals were fed fresh tubers, vegetables, fruit and Pronutro cereal supplement (Bokomo Food Products, Namibia) ad libitum.

Wild animals

Wild-caught animals used in this study included:

10 Adult Cryptomys hottentotus mahali (C.h.m.; 103.8 ± 6.2g), 4 female and 6 males
9 Adult Cryptomys hottentotus pretoria (C.h.h.; 113.1 ± 9.2g), 6 females and 4 males
10 Adult Cryptomys hottentotus hottentotus (C.h.h.; 88.5 ± 5.2g), 1 female and 9 males
10 Adult Georychus capensis (G.c.; 155.0 ± 17.8g), 5 females and 5 males

Cryptomys hottentotus mahali, Cryptomys hottentotus pretoriae, Georychus capensis, and Cryptomys hottentotus hottentotus were captured using humane live Hickman traps which comprise of a plastic tunnel trap with a door that is triggered as the animal feeds on sweet potato. The traps are positioned at the entrance of the tunnel and covered in soil so that they remain cool and the mole-rat detects no air currents.

The species used in the study were all adult animals and the necessary ethics clearance from the University of Pretoria and capture permits were obtained from the relevant nature conservation authorities in the Western Cape and Gauteng provinces of South Africa.

Once captured animals were housed in plastic containers of 30cmx60cmx30cm during capture and were transported back to the laboratory in IATA approved containers with temperature control to 25oC by air conditioning in the vehicle. All mole-rats used for the experiment were euthanized at the University of Pretoria by trained personnel. Animals had to be euithanized by decapitation for other studies and the BAT was opportunistically harvested.

Field-collected samples	Mole-rats (other than naked mole-rats) were housed in temperature controlled rooms at 25oC under a 12L:12D photoperiod and were fed chopped sweet potato, apples, carrots and sweet corn. Mole-rats obtain all their water requirements from the food source as in the natural habitat where they feed upon underground storage organs such as bulbs, corms and tubers. Solitary species were housed on their own and social species were housed as colony units. The animals were maintained in plastic crates 30cmx60cmx30cm provided with wood shavings and paper towels as nesting. Animals were fed daily and cleaned weekly. Following experimental hypoxic or normoxic exposures, all CHM, CHH, CHP, and GC were rapidly killed for tissue collection.
Ethics oversight	All experimental procedures were approved by the University of Ottawa Animal Care Committee (protocol #2535), in accordance with the Animals for Research Act and by the Canadian Council on Animal Care. Trapping and experiments conducted in South Africa were conducted under appropriate permits issued by Cape Nature Conservation and the Department of Nature Conservation in the Western Cape, Republic of South Africa (CN44-31-2285) and with experimental procedures approved by the animal ethics committee of the University of Pretoria (EC069-17).

Note that full information on the approval of the study protocol must also be provided in the manuscript.
